# Posterior annulus shortening increases leaflet coaptation in ischemic mitral incompetence: a new and valid technique

**DOI:** 10.1186/1749-8090-8-S1-O276

**Published:** 2013-09-11

**Authors:** R Hetzer, A Amiri, N Solowjowa, H Siniawski, E Delmo Walter

**Affiliations:** 1 Cardiothoracic Surgery, Deutsches Herzzentrum Berlin, Berlin, Germany

## Background

We introduce a valid concept and strategy of a posterior annulus shortening to augment leaflet coaptation in ischemic mitral incompetence (IMI), and report its long-term outcome.

## Methods

The technique consists of posterior annulus shortening reducing the annular diameter between 23 and 25 mm and decreasing its orifice to between 3.5 to 4.5 cm2. This size is sufficient to ensure an adequate leaflet coaptation area. The shortened annulus is reinforced by an untreated autologous pericardial strip. This augments the posterior leaflet by increasing the ratio of leaflet area/valve orifice where the gap in coaptation is the greatest. The area which the posterior leaflet offers to the anterior leaflet for coaptation during closure is heightened by the tissue strip, making valve closure possible even in advanced leaflet restriction.

## Results

Between 1992 and 2012, 75 patients (mean age 64.56±10.37,range 35.0-86.1, years) underwent posterior annulus shortening to augment leaflet coaptation surface area in repair of IMI. At a mean follow-up of 7.62±0.66 (range 3.6-20.9) years, NYHA functional class significantly improved, LVEFF increased and there was a tremendous abatement of MI (p<0.01). Annular area was reduced from 9.2 cm2 to 5.8 cm2. From a complete absence of coaptation area, it creased to 6.6 mm after the repair. CT showed posterior annulus size reduction from 70.4 mm to 64 mm and an increase in posterior leaflet length from 15.92 to 19.6 mm. A most remarkable CT finding was the increase in coaptation length from 5.2 to 8.2 mm. Freedom from reoperations were 95.3±2.7% and 86.4±5.6%, and survival rates were 71.9±5.5%, and 65.1±6.3% at 1and 18 years, respectively. Freedom from moderate or greater MI is 80.7±9 % at 10 years.

## Conclusion

Posterior shortening annuloplasty with pericardial strip augmentation addressing the lack of leaflet coaptation, is a simple and highly effective repair technique to restore valve competence in ischemic MI.

